# Efficiency of CAR-T Therapy for Treatment of Solid Tumor in Clinical Trials: A Meta-Analysis

**DOI:** 10.1155/2019/3425291

**Published:** 2019-02-11

**Authors:** Bin Hou, Yao Tang, Wenhan Li, Qingnuo Zeng, Dongmin Chang

**Affiliations:** ^1^Department of Surgical Oncology, The First Affiliated Hospital of Xi'an Jiaotong University, Xi'an, Shaanxi, China; ^2^Department of General Surgery, Xi'an No. 3 Hospital, Xi'an, Shaanxi, China

## Abstract

**Background:**

Chimeric antigen receptor T (CAR-T) cell therapy has achieved unprecedented success among hematologic tumors, but its role in treating solid tumors is still unclear.

**Methods:**

A comprehensive search of electronic databases up to June 1, 2018, was carried out by two independent reviewers. We included studies which focused on the association between CAR-T cell therapy and patient response rate and survival time in solid tumors.

**Results:**

22 studies with 262 patients were included in our meta-analysis. The overall pooled response rate of CAR-T cell therapy was 9% (95% confidence interval (CI): 4-16%). Subgroup analysis (analyses) demonstrated that CAR-T therapy could perform its best therapeutic effect on neuroblastoma, while barely works among gastrointestinal malignancies. Moreover, the treatment efficacy was not significantly impacted by different treatment strategies (lymphodepletion before T cell infusion, transfection method, cell culture duration, persistence of CAR-T cells, transfection efficacy, total cell dose, and administration of IL-2). Only T cell culture duration was associated with better clinical prognosis.

**Conclusions:**

Although CAR-T cell therapy did not have satisfactory responses in solid tumors, researchers were still holding an optimistic attitude towards its future efficacy with more modifications of its structure.

## 1. Introduction

With the rapid development of molecular biology, the concept of cancer treatment makes great progress. Chimeric antigen receptor T (CAR-T) cell therapy, whose initial conceptualization was put forward in the late 1980s, has been approved by FDA in 2017 as the first genetically engineered cellular treatment for pediatric and young adult acute lymphoblastic leukemia (ALL) [1]. This therapy, in theory, allows CARs, which are artificially engineered receptors that could express on cell surface with non-HLA-restricted tumor antigens, to activate T cells and guide them specifically to tumor cells to perform their function. For now, CD-19 is the most attractive target in this immunotherapy. Encouragingly, T cells expressing the CD19-CARs have achieved unprecedented therapeutic efficacy in malignant hematological diseases with up to 90% complete remission rate in ALL and more than 60% in non-Hodgkin's lymphoma (NHL) [2]. In a phase II trial conducted by Neelapu et al. [3], 111 patients with B cell lymphoma were recruited and they accepted anti-CD19 CAR-T cell therapy. The objective response rate and the complete response rate were 82% and 54%, respectively.

Enlightened by the idea of adoptive immunotherapy and its great success in treating hematological malignancies, a number of preclinical CAR-T cell therapy trials have been carried out in solid tumors. However, the results were variable in different tumors with different therapeutic strategies. Louis et al. [4], for example, used CAR-T cells in treating neuroblastoma. They found that 4 out of 19 (52.9%) patients achieved objective clinical responses and 3 of them even got complete remission. O'Rourke et al. [5], however, treated recurrent glioblastoma patients with anti-EGFRvIII CAR-T cells. None of the 10 patients has positive response (partial response or complete response) to this therapy. Although the results were unsatisfactory, researchers still believed that CAR-T cell therapy was a promising method for tumor treatment.

Owing to the variability of those clinical trials, it is extremely necessary to analyze the impact of CAR-T therapy on tumor treatment collectively. Currently, there are three meta-analyses concerning the efficacy and safety of CAR-T cell therapy in hematological malignancies [6–8]. Solid tumor treatment efficacy, however, has no satisfactory synthesis data yet. Thus, we conducted this systemic review and meta-analysis to comprehensively investigate the treatment efficacy of CAR-T cell therapy in solid tumors. We also used subgroup analysis to explore the factors that could affect the efficacy of this therapy. Our team focused on the evaluation of the clinical outcomes of different treatments [9]. Therefore, we hoped our results could help researchers and clinicians in clinical trial design.

## 2. Materials and Methods

### 2.1. Data Sources

A comprehensive search in the PubMed database up to June 1, 2018, was undertaken using a combination of the following keywords: “CAR-T therapy,” “chimeric antigen receptor T cell,” “solid tumor,” and “prognosis.” Meanwhile, abstracts from the American Society of Clinical Oncology (ASCO) using the same search terms were evaluated. An independent search of the Embase database was also carried out.

### 2.2. Study Selection

The following criteria were considered in this research: (1) prospective or retrospective cohort studies of patient with nonhematologic solid tumors and (2) assessment of the prognostic effect of CAR-T therapy on complete response rate or partial response rate, overall survival (OS), progression-free survival (PFS), and alive with disease (AWD). Articles were excluded with any of the following criteria: (1) patient with hematologic malignancies; (2) animal experiments and non-English studies; (3) duplicated data; (4) comments, reviews, or meta-analyses without original data; and (5) no clinical outcome.

### 2.3. Data Extraction

Two independent investigators reviewed eligible articles and extracted data from studies. Gender, age, type of solid tumor, gene transduction method, T cell culture time, original T cell sources, lymphodepletion, IL-2 administration for patient, total infused CAR^+^ T cell number (data in cells/kg or cell/m^2^ were multiplied by 60 kg or 1.73m^2^, respectively), CAR^+^ T cell persistence time, and clinical outcomes of patients with CAR-T therapy were collected from each study. The primary endpoint was the response rate of CAR-T immunotherapy in this meta-analysis. Therapy response was defined by the RECIST 1.1 and immune-related response criteria.

### 2.4. Statistical Analysis

All statistical analysis was performed by the R software version 3.3.0 (http://www.r-project.org). All statistical tests were two-sided, and *P* ≤ 0.05 was considered statistically significant. The heterogeneity of data among included studies was examined by using Cochran's *Q*-test and Higgins' *I*^2^ statistic. We defined insignificant heterogeneity if the *I*^2^ value was <50% and we used a fixed-effect model to calculate the parameters. If *I*^2^ value is ≥50%, we discerned that a significant heterogeneity existed and a random-effect model was applied to the data. Subgroup analyses were conducted according to tumor sites (gastrointestinal tumor, hepatobiliary and pancreatic tumor, neurologic tumor, and other tumors), lymphodepletion before T cell infusion (yes or no), transfection method (by lentivirus or retrovirus), cell culture duration (<14 days vs. ≥14 days), persistence of CAR-T cells (<4 weeks vs. ≥4 weeks), transfection efficacy (<50% vs. ≥50%), total cell dose (<10^9^ vs. ≥10^9^), and administration of IL-2 (yes or no). Assessment of publication bias was examined by the use of funnel plots and confirmed by Egger's and Begg's test.

## 3. Results

### 3.1. Study Characteristics

**Figure 1:**
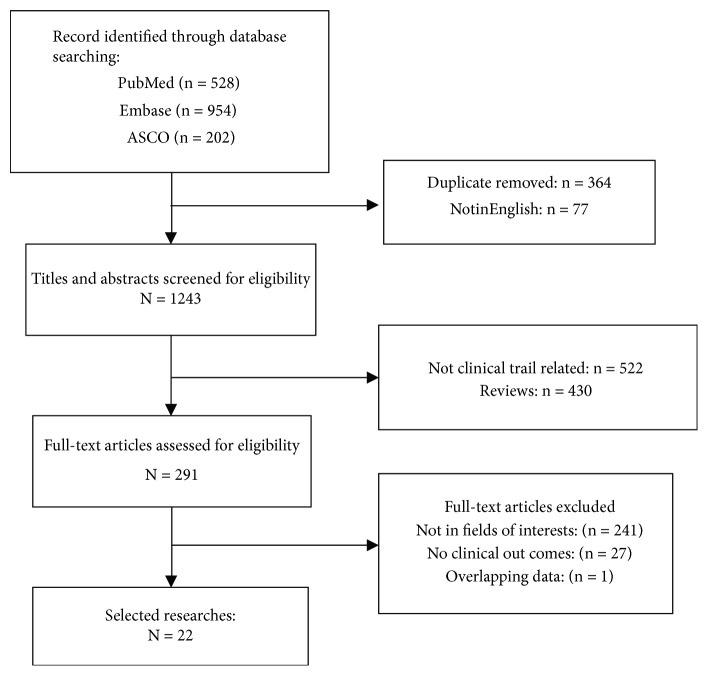
Flow diagram of study selection process.

Publications were initially identified following our search strategy. After removing duplicated publications, 1243 studies were selected for further analysis. We next screened the title and abstract of each article. Reviews, in vitro studies, and nonhuman studies were excluded from our analysis. Consequently, 291 eligible studies received full-text evaluation. Among them, 27 studies with insufficient survival data were excluded from this analysis. Thus, a total of 22 studies with 262 patients fulfilled our inclusion criteria and were enrolled in our study; only 82 patients from 8 clinical researches had OS data. The selection process is shown in Figure 1.

Table 1 summarizes the baseline characteristics of included studies. Of all eligible studies, five studies reported on neurological tumors (*n* = 7) [4, 5, 10–14], followed by hepatobiliary and pancreatic cancers (*n* = 5) [15–19], melanoma and sarcoma (*n* = 4) [20–22], gastrointestinal malignancies (*n* = 2) [23, 24], prostate cancer (*n* = 1) [25], breast cancer (*n* = 1) [26], non-small-cell lung cancer (*n* = 1) [27], and others [28]. Most of the patients enrolled in this analysis had advanced tumor (stages III-IV). There were two main transfection methods (lentivirus and retrovirus) with various cancer-specific antigens (EGFR, CEA, HER2, etc.) used in these studies. All of the patients were infused with autologous T cells. 134 (51.1%) patients received lymphodepletion before CAR-T cell infusion and 96 (36.6%) patients had IL-2 treatment during CAR-T therapy.

### 3.2. Treatment Response

**Figure 2:**
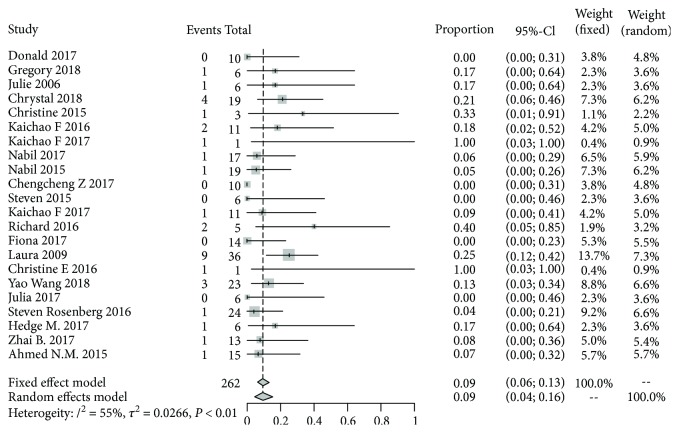
Forest plot for response rates and confidence intervals in each study and the overall.

The response rate of CAR-T cell therapy in each study varied, ranging from 0% to 100%. Six patients (2.3%) got complete response and 25 patients (9.5%) achieved partial remission. As shown in Figure 2, the overall remission rate of 9% (95% CI: 4%-16%) was observed in a random-effect model with significant heterogeneity among studies (*I*^2^ = 55%, *P* < 0.01).

**Figure 3:**
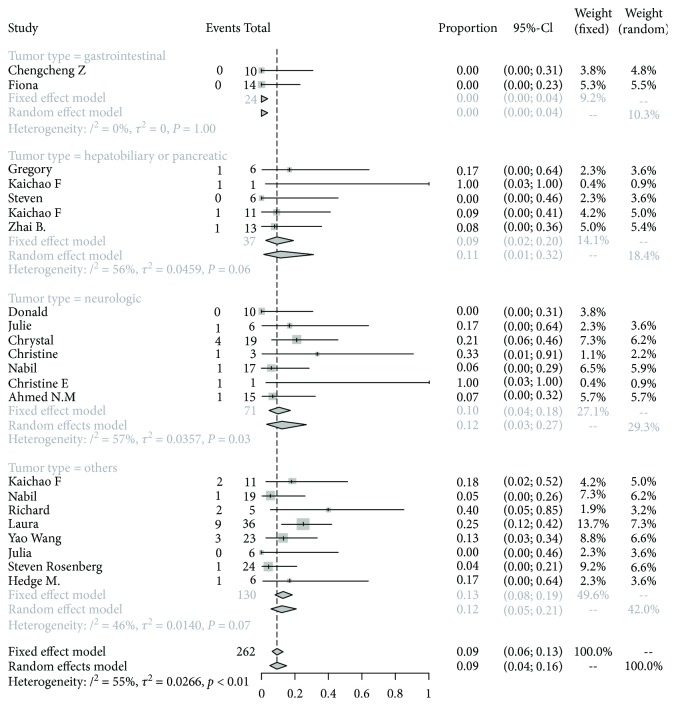
Forest plot for response rates and confidence intervals among different tumor sites.

Subgroup analyses were carried out according to tumor sites (gastrointestinal tumor, hepatobiliary and pancreatic tumor, neurologic tumor, and other tumors), lymphodepletion before T cell infusion (yes or no), transfection method (by lentivirus or retrovirus), cell culture duration (<14 days vs. ≥14 days), persistence of CAR-T cells (<4 weeks vs. ≥4 weeks), transfection efficacy (<50% vs. ≥50%), total cell dose (<10^9^ vs. ≥10^9^), and administration of IL-2 (yes or no). Our results demonstrated that the main source of heterogeneity in our analyses derived from different tumor site (*P* = 0.0034). As shown in Figure 3, the pooled response rate reached 11% (95% CI: 1%-32%, *I*^2^ = 56%) in hepatobiliary and pancreatic cancer, 12% (95% CI: 3%-27%, *I*^2^ = 57%) in neurologic tumor, and 12% (95% CI: 5%-21%, *I*^2^ = 46%) in other tumors. Notably, CAR-T cell therapy showed frustrated treatment effect among gastrointestinal cancer. We did not find significant heterogeneity within the rest of subgroups (all *P* > 0.05), but the *I*^2^ statistic for heterogeneity was high in each group, indicating the heterogeneous trend in each study (Table 2).

**Figure 4:**
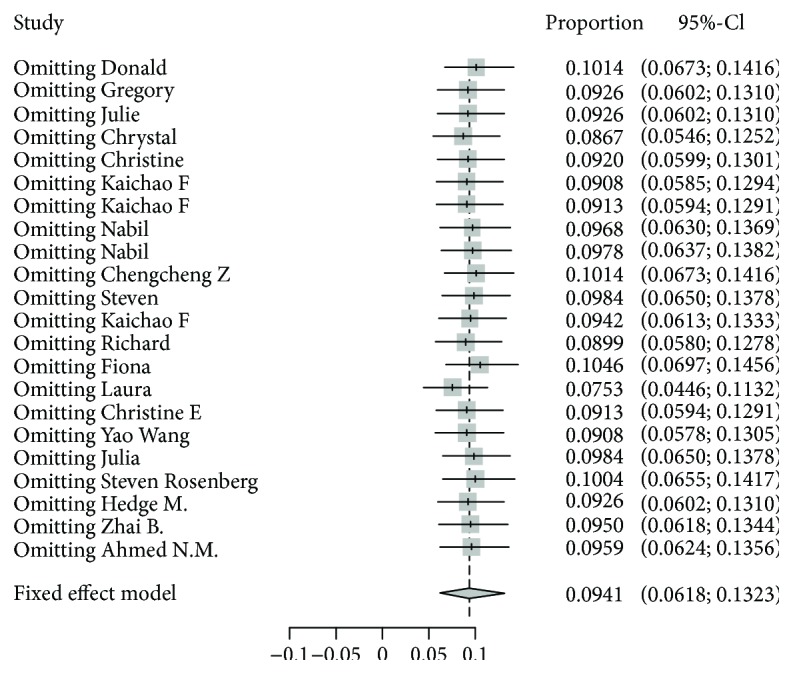
Sensitivity analysis using a “one-study removed” model.

**Figure 5:**
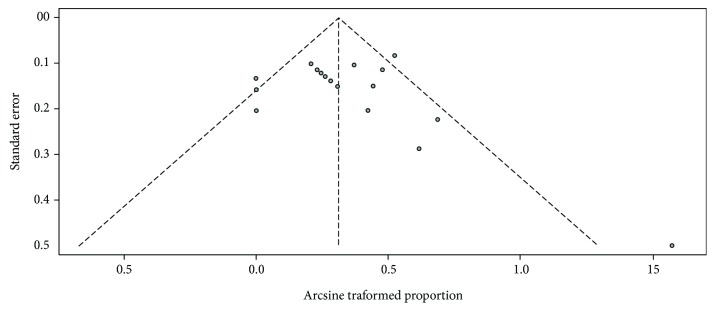
Funnel plot of publication bias in the meta-analysis.

Sensitivity analysis using a “one-study removed” model was conducted to testify the stability of our result. As shown in Figure 4, the observed effect size of response rate was not significantly affected by removing a single study each time. Publication bias was not found by visual inspection of the funnel plot (Figure 5) and confirmed by Begg's test and Egger's tests (all *P* > 0.05). Thereby, the reliability of our results could be confirmed.

### 3.3. Patients' Prognosis

**Figure 6:**
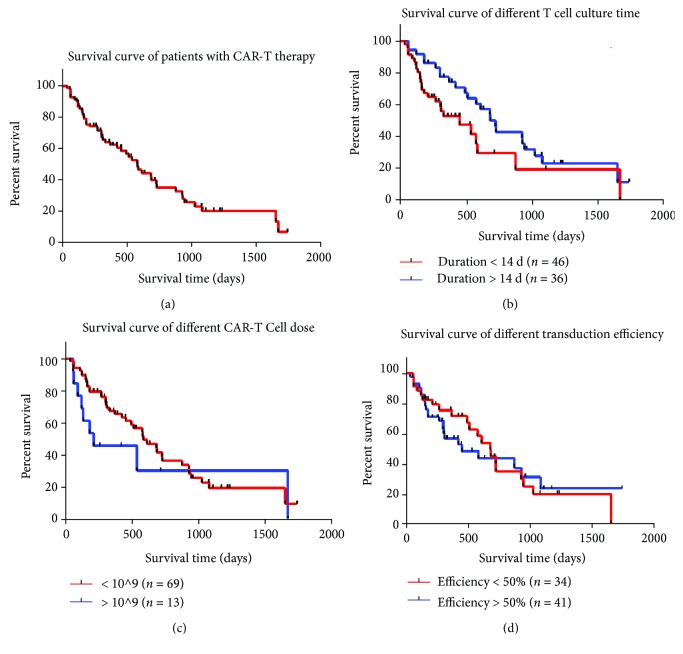
Overall survival (OS) curves. (a) Survival curve of patients with CAR-T therapy. (b) Survival curve of different T cell culture time. (c) Survival curve of different CAR-T cell dose. (d) Survival curve of different transduction efficiency.

The median survival time of patients with CAR-T therapy (*n* = 82) was 576 days. The one-year and three-year OS rates were 64.1% and 20.4%, respectively (Figure 6(a)). Then we compared clinical outcomes of patients who accepted different CAR-T therapies. We divided the 82 patients into different groups according to total T cell dose, transduction efficiency, and T cell culture duration. The results showed that only T cell culture duration was associated with better clinical prognosis. As shown in Figure 6(b), patients with T cell culture duration more than 14 days (*n* = 36) had better treatment outcomes than those with T cell culture duration less than 14 days (*n* = 46) (median survival time: 723 days vs. 451 days, *P* = 0.02). Of note, patients with lower infused CAR^+^ T cell dose tend to have better prognosis than those with higher T cell dose, although the result is insignificant (584 vs. 210 days, *P* = 0.17) (Figure 6(c)). The survival curve of patients with different transduction efficiency is shown in Figure 6(d).

## 4. Discussion

CAR-T cell therapy opens a new era for cancer treatment by using artificial modified autologous immune cells. Compared to previous conservative treatments, such as radiotherapy and chemotherapy, this therapeutic approach has the ability to recognize unique antigen expressed in the tumor surface (like a monoclonal antibody) and subsequently induces full T cell activation. By doing this, cancer cells are eliminated specifically while normal somatic cells are well preserved. To date, this approach has made a great success in treating hematological malignancies. However, its therapeutic effect in solid malignancies is unsatisfactory. Although several small-size clinical trials have been made, there is no systematic review to evaluate its efficacy and safety. In this meta-analysis, with well-designed selection method and strictly statistical analysis, we concluded that the response rate of CAR-T cell therapy among solid tumors reaches 9% (95% CI: 4%-16%). Moreover, we observed that the best therapeutic effect of CAR-T cell therapy on advanced cancers was observed in neuroblastoma (response rate: 33%, 95% CI: 1%-91%). Although the outcome was a little bit frustrating, it was still too early to draw a conclusion of its efficacy in solid tumors. There are still great potentials for us to tap.

The formation of solid tumors is different from that of hematological tumors; therefore, certain obstacles should be taken into consideration when clinicians are trying to transfer CAR-T cell therapy from liquid cancers to solid tumors. Unlike hematological tumors, solid tumor cells are rarely present in the circulation system, but tumor cells could form several discrete foci where T cells are obviously difficult to infiltrate. In addition to the physical barriers, chemokines that are secreted by solid tumor cells are usually abnormal, which may lead to insufficient T cell recruitment [29]. To address this issue, scientists try to infuse CAR-T cells by intratumoral routes and have achieved relatively satisfactory results with acceptable toxicity in a mouse model [30]. Moreover, some molecular modifications on T cells have been made to enhance the efficacy of T cell recruitment [16, 31–33]. After T cells infiltrated the tumor, intratumoral T cells usually cannot perform their antitumor function very well. Two checkpoint molecules, PD-1 and CTLA-4, particularly contribute to this phenomenon. A number of tumor cells could express the ligand of those two molecules and consequently lead to immune activation suppression [34–36]. To solve this problem, researchers attempt to combine CAR-T cell therapy with immune checkpoint blockade [37–39]. Although all of those methods are still in preclinical trials, some of them may lead to meaningful improvement for CAR-T cell therapy with solid malignancies.

Safety is another vital criterion to evaluate a novel cancer-treating strategy. In CAR-T cell immunotherapy, on-target off-tumor toxicities and cytokine-released syndrome (CRS) are the two major adverse events. Because most of the solid tumors have epithelial origin, it is difficult to find an antigen specifically expressed in the tumor cell surface while being absolutely absent in normal epithelial cells. Therefore, off-target toxicities happened. The main character of toxicity is tissue or organ damage induced by the overflow of inflammatory cytokines. The symptoms of CRS are more variable and some of them could be long-lasting and life-threatening. The underlying mechanism of CRS is still ambiguous. Except for clinical examination markers used in clinical practice, different host status and tumor microenvironment may contribute to the occurrence of CRS. Many organs and systems in our body, like the circulatory system, respiratory system, digestive system, and nervous system, get involved in those adverse events. Some articles [17, 22, 24, 27] included in our analysis found that the toxicities may positively correlate with infusion dosage, but the adverse effect is acceptable for the majority of them and they could be alleviated by medical interference. However, severe adverse effects (grade 3 and grade 4) were reported in 80% (12/15) of the included articles. Most of them were neurotoxicity (such as fatigue, dizziness, dysgeusia, and headache) and nonspecific symptoms (fever and muscle aches, etc.). Feng et al. [17] reported that a patient with advanced cholangiocarcinoma broke out severe complications after CAR-CD133 T cell infusion, including an intermittent upper abdominal dull pain, chills, fever, and rapidly deteriorative grade 3 systemic subcutaneous hemorrhages and congestive rashes together with serum cytokine release, which needed emergent medical intervention including intravenous methylprednisolone. Our analysis also showed that patients with lower infused CAR^+^ T cell dose tend to have better prognosis than those with higher T cell dose (584 vs. 210 days, *P* = 0.017). We should fully notice that enhancing treatment dosage may also increase the risk of experiencing an adverse drug reaction. Collectively, most complications could be attenuated successfully by standard supportive therapy, but any benefit of CAR-T cell therapy must be weighed against the risk of severe or even life-threatening complications. The toxicities and optimum therapeutic dosage remain a topic for further investigation.

Certain limitations should be taken into consideration. Firstly, CAR-T cell therapy for advanced solid tumors is a brand-new treatment strategy; for now it lacks large-scale clinical data to comprehensively evaluate its efficacy as well as safety. The main data in our analysis only contains response rate and short-term survival rate, whether patients could benefit from this treatment method is still uncertain. Furthermore, evaluation standards for adverse events are variable in each study; we only did descriptive analysis instead of statistical analysis in this part so that the result is inaccurate. There is still much work to be done before its wide clinical application.

## 5. Conclusions

Our meta-analysis indicates that the pooled response rate of CAR-T cell therapy in solid tumors reaches 9%. Although we have achieved some encouraging results, the construction improvement of CAR-T cells is still needed to fully recruit and activate immune cells to eliminate its target. There is still a long way to go before its application in clinical practice. However, by combining this new treatment method with updating technologies and lessons from retrospective studies, we believe CAR-T cell therapy will perform better clinical efficacy in treating solid tumor malignancies.

## Figures and Tables

**Table 1:** The baseline characteristics of eligible clinical researches.

Author	Country	No.	Vector	T cell origin	Cell culture	Transfection method	T cell treatment	T cell persistence	Diagnosis	Target	Transduction efficiency	Lymph depletion	IL-2 for patient	T cell dose	Response	Ref
Donald (2017)	USA	10	CD8+CD3*ζ*	Autologous	NA	Lentiviral	Irradiated+IL-2	1 day	Glioblastoma	EGFRvIII	19.75%	No	No	1∗10^8^~5∗10^8^	9∗SD, 1∗PD	[5]
Gregory (2018)	USA	6	CD3*ζ*+CD28	Autologous	8~12 days	Lentiviral	NA	1 day	Pancreatic carcinoma	Mesothelin	90.7% (76.9~95.9%)	No	No	1~3∗10^8^/m^2^	5∗SD, 1∗PR	[14]
Juiie (2006)	USA	6	CD4+CD3	Autologous	14 days	Lentiviral	OKT3+IL2+irradiated	7 days	Neuroblastoma	CE7R	NA	Yes	No	10^8^~10^9^/m^2^	1∗PR, 5∗PD	[13]
Chrystal (2018)	USA	19	14.G2a-z	Autologous	18~23 days	Retroviral	OKT3+IL2+irradiated	6 weeks	Neuroblastoma	GD2+EBV+	NA	No	No	2∗10^7^~1∗10^8^/m^2^	8∗NED, 3∗C, 4∗PD, 1∗PR, 1∗SD, 2∗tumor nuosis	[4]
Christine (2015)	USA	3	CD4+CD3+GM-CSF	Autologous	14 days	Cytomegalovirus	OKT3+IL2+irradiated	>6 weeks	Glioblastoma	IL13R*α*2	>75%	Yes	No	96~10.6∗10^8^	2∗SD, 1∗PR	[12]
Kaichao (2016)	China	11	CD137+CD8+CD3*ζ*	Autologous	10~13 days	Lentiviral	IL-2	>4 weeks	NSCLC	EGFR	29.28% (20.87~31.22%)	Yes	Yes	0.45~1.09∗10^7^	5∗SD, 4∗PD, 2∗PR	[26]
Kaichao (2017)	China	1	CD137+CD3*ζ*	Autologous	2 days	Lentiviral	IL-2	2 months	Cholangiocarcinoma	EGFR+CD133	NA	No	No	2.2∗10^6^/kg	PR	[16]
Nabil (2017)	USA	17	FRP5+CD28	Autologous	3~4 weeks	Retroviral	NA	1 year	Glioblastoma	HER2	39%	No	No	10^6^~10^8^/m^2^	1∗PR, 7∗SD, 9∗PD	[9]
Nabil (2015)	USA	19	FRP5+CD28	Autologous	10~21 days	Retroviral	IL-2+OKT3	>6 weeks	Sarcoma	HER2	65.2% (36.2~88%)	No	No	1∗10^4^~1∗10^8^/m^2^	12∗PD, 4∗SD, 1∗PR, 2∗NE	[19]
Chengcheng (2017)	China	10	IgG4+CD28+CD3z	Autologous	14~16 days	Lentiviral	IL-2	4~6 weeks	Colorectal cancers	CEA	33.7% (14.7%~43.2%)	Yes	No	1∗10^5^~1∗10^8^/kg	8∗SD, 2∗PD	[23]
Steven (2015)	USA	6	CD28+CD3*ζ*	Autologous	10~14 days	Retroviral	OKT3+IL-2	NA	Liver metastases	CEA	45% (10%~64%)	No	Yes	1∗10^8^~1∗10^10^	1∗SD, 5∗PD	[14]
Kaichao (2017)	China	11	CD137+CD3*ζ*	Autologous	12 days	Lentiviral	OKT3+IL-2+IFN	4 weeks	Biliary tract and pancreatic cancers	HER2	9.9% (5.5%~11.4%)	Yes	No	2.1∗10^6^/kg (1.4~3.8∗10^6^/kg)	1∗PR, 5∗PD, 5∗SD	[17]
Richard (2016)	USA	5	—	Autologous	14 days	Retroviral	OKT3+IL-2	3 weeks	Prostate cancer	PSMA	5~56%	Yes	Yes	10^9^~10^10^	2∗PR, 2∗NR, 1∗MR	[24]
Fiona (2017)	UK	14	CD3*ζ*+MFE*ζ*	Autologous	7 days	Retroviral	OKT3+IL-2	14 days	Gastrointestinal malignancies	CEACAM5	26.9% (20.1~37.3%)	Yes	Yes	10^9^~5∗10^9^	7∗SD, 7∗PD	[25]
Laura (2009)	USA	20	TCR-*β*+TCR-*α*	Autologous	9~12 days	Retroviral	OKT3+IL2+irradiated	4 weeks	Metastatic melanoma	DMF5	71% (33~95%)	Yes	Yes	2~107∗10^9^	14∗NR, 6∗PR	[21]
		16	TCR-*β*+TCR-*α*	Autologous	9~12 days	Retroviral	OKT3+IL2+irradiated	4 weeks	Metastatic melanoma	Gp100	82% (55~97%)	Yes	Yes	2.3~110∗10^9^	13∗NR, 1∗CR, 2∗PR	[21]
Christine E.	USA	1	4-1BB+CD19	Autologous	<14 days	Lentiviral	rhIL-2+rhIL-15	<4 weeks	Glioblastoma	IL13R*α*2	74%~90%	No	No	1.44∗10^8^	1∗CR	[11]
Yao Wang	China	23	CD137+CD3z	Autologous	<14 days	Lentiviral	IL-2	4 weeks	HCC, PC, CRC	CD133	11.23%~56.47%	No	No	0.5~2∗10^6^/kg	3∗PR, 14∗SD, 6∗PD	[27]
Julia	USA	6	CD3*ε*	Autologous	8~12 days	Electroporation	NA	<14 days	Breast cancer	c-Met	36.5%~85.8%	No	No	3∗10^7^~3∗10^8^	1∗SD, 2∗PD, 3∗DOD	[25]
S. Rosenberg	USA	24	CD3	Autologous	NA	Retroviral	NA	NA	Metastatic melanoma and renal cancer	VEGFR2	NA	Yes	Yes	10^6^~3∗10^10^	1∗PR, 1∗SD, 22∗PD	NCT 01218867
Hegde M.	USA	6	CD28	Autologous	NA	NA	NA	6 weeks	Sarcoma	HER2	NA	Yes	NA	1∗10^8^/m^2^	1∗CR, 2∗SD, 3∗PD	[20]
Zhai B.	China	5	NA	NA	NA	Lentiviral	NA	NA	Hepatocellular	GPC3	NA	No	NA	0.92∗10^7^~8.72∗10^7^/kg	5∗PD	[18]
		8	NA	NA	NA	Lentiviral	NA	NA	Carcinoma	GPC3	NA	Yes	NA	0.013∗10^7^~14.68∗10^7^	1∗PR, 3∗SD, 2∗PD	[18]
Ahmed N. M.	USA	15	CD28.zeta	Autologous	NA	NA	NA	12 weeks	Glioblastoma	HER2	67% (46%~82%)	No	NA	1∗10^6^~1∗10^8^/m^2^	1∗PR, 4∗SD, 10∗PD	[10]

SD: stable disease; PD: progressive disease; PR: partial response; CR: complete response; NED: no evidence of disease; NR: no response; MR: minor response; NE: not evaluable.

**Table 2:** Pooled response rate for CAR-T cell therapy according to subgroup analyses.

Subgroups	Studies	Pooled RR	95% CI	Heterogeneity (*I*^2^)	*P* value between groups
*Lymph depletion*					
Yes	11	11%	4%-20%	54%	0.638
No	11	8%	1%-19%	62%	
*Transfection method*					
Lentiviral	11	10%	3%-21%	56%	0.935
Retroviral	7	9%	2%-22%	69%	
*Cell culture duration*					
<14 days	11	11%	3%-23%	67%	0.806
≥14 days	6	13%	3%-28%	50%	
*Persistence of CAR-T cells*					
<4 weeks	10	12%	3%-25%	69%	0.777
≥4 weeks	9	10%	3%-19%	41%	
*Transfection efficacy*					
<50%	10	10%	1%-16%	62%	0.335
≥50%	7	14%	3%-30%	61%	
*Total cell dose*					
<10^9^	13	11%	4%-19%	49%	0.939
≥10^9^	7	11%	2%-27%	66%	
*Administration of IL-2*					
Yes	6	11%	1%-29%	70%	0.827
No	12	9%	3%-20%	60%	

## Data Availability

The data used to support the findings of this study are included within the article.
